# Anomalous cyclic in the neutrino oscillations

**DOI:** 10.1038/s41598-023-39871-3

**Published:** 2023-08-04

**Authors:** E. Aydiner

**Affiliations:** Department of Physics, Faculty of Science, Istanbul University, 34134 Istanbul, Turkey

**Keywords:** Experimental particle physics, Phenomenology, Particle physics, Quantum physics

## Abstract

Neutrino physics is one of the most important topics in particle physics and cosmology. As it is known, neutrinos are weakly interacting fundamental particles with chargeless and very small masses. One of the most characteristic features of the neutrino that make a difference from other elementary particles is that it oscillates between the mass and flavour eigenstates. Due to these oscillations, neutrinos change from one flavour to another. So far in theory the possible effects of deformed space-time effects on oscillation have not been considered. In this study, we show for the first time that a deformed space-time metric will lead to fractional dynamics between mass and flavour changes and therefore cause a phase shift in the oscillation period. We also shortly discuss the possible relation between anomalous cyclic and relic neutrinos. The modification in the oscillation probabilities due to the studied effect in this work could be seen using relic neutrinos.

## Introduction

In recent 90 years, neutrino physics has been one of the most curious and hotly debated topics in particle physics and cosmology. We are faced with many unsolved problems in this area yet. Without understanding the mystery behind neutrinos, it seems impossible to understand their importance and place in particle physics and cosmology.

Historically, the neutrino $\nu$ was proposed in December 1930 by Pauli to explain the continuous energy spectrum of the electrons measured in $\beta$-decays. Pauli named these particles neutrons because of their uncharged nature. However, after Chadwick’s discovery of the heavier and uncharged particle in 1932, Fermi changed this name to the neutrino.

In the 1950s, Pontecorvo proposed the massive neutrinos for the first time. Afterwards, in 1957 he suggested a practical method for investigating neutrino oscillations in the vacuum setting, an analogy with kaon oscillations^1–3^. In Pontecorvo’s scenario, mixing the flavor and mass eigenstates of neutrinos leads to oscillation. This model imposes that the neutrinos have a tiny mass, unlike the standard model of particle physics. As we will show later, each type of oscillation is completely periodic, although the periods of neutrino oscillations vary depending on the source. The theoretically proposed neutrino oscillation has also been observed experimentally^4,5^.

It is known that neutrinos are left-handed fundamental particles like quarks, photons, and electrons and they are generated by weak interactions $W^{\pm }$and $Z^{0}$ which are carriers of the force between fermions. They cannot decay into other particles and do not carry an electric charge. Therefore, it is challenging to detect neutrinos experimentally. However, using unusual experimental setups, in 1956, the electron neutrino $\nu _{ e}$ in the inverse $\beta$ decay was first discovered by Reines and Cowan^6^. This pioneering discovery was awarded the Nobel Physics Prize in 1995. In 1962, the muon neutrino $\nu _{ \mu }$ interactions were detected by Lederman et al.^7^. They shared Nobel Prize in 1988 due to the neutrino beam method and the demonstration of the doublet structure of the leptons through the discovery of the muon neutrino. Finally, the tau particle was detected in a series of experiments between 1974 and 1977 by Perl with his colleagues at the SLAC–LBLgroup^8^. Perl shared the 1995 Nobel Prize in Physics with Reines for the discovery of the tau neutrino. Additionally, in 2000, the interactions of tau neutrino $\nu _{ \tau }$ were first confirmed by the DONUT collaboration at Fermilab^9^.

Solar neutrino problem was noticed in 1960’s that remained unsolved for nearly three decades. However, to explain this inconsistency, it has been suggested that neutrinos may have mass and oscillate between flavours. In the following years, solar and cosmic neutrinos were investigated. In the 1960s, the flux of electron neutrinos coming from the sun in the Homestake experiment and a discrepancy between the experiment’s results and the predictions of the Standard Model was found^10^. This discrepancy was called the solar neutrino problem. Furthermore, Koshiba also studied solar and cosmic neutrino detection. Particularly, he observed neutrinos from the SN 1987A supernova in the nearby Large Magellanic Cloud^11,12^. Therefore, Davis and Koshiba shared Nobel Physics Prize in 2002 for detecting neutrinos coming from the sun and SN 1987A supernova.

Many experimental studies have been conducted to understand the solar neutrino problem and confirm the neutrino oscillation, such as the Sudbury Neutrino Observatory (SNO) and Super-Kamiokande. In 1998, the difference of squared mass of the neutrinos was measured and detected that neutrinos oscillate from one flavour to another^4^. On the other hand, the first experimental evidence of neutrino oscillation was published in 2001 by SNO^5^. Experimental findings revealed that neutrinos coming from the sun oscillate. These results also supported that neutrinos would have mass. Kajita and McDonald received the 2015 Nobel Prize for Physics.

As a result, it is experimentally shown that they have a tiny mass compared to other elementary particles^4,5,13–16^. Neutrino masses are so small that no experiment has succeeded in measuring them so far. It is assumed that the masses of all fundamental particles come from the Higgs field, but neutrino might get their masses another way. Since the mass of each neutrino is unknown, the upper bound on the total mass of the three neutrinos is obtained from cosmological calculations. Accordingly, the total mass of the three neutrinos should not exceed 50 eV^17^. Some research results indicate that the neutrino’s mass should be about one million times less than the mass of the electron^15,16,18,19^.

Today, we know that many natural processes create neutrinos, such as artificial nuclear reactions, nuclear reactions in stars, particle decay processes, supernova explosions, and neutron star spin-down. However, these particles are the most abundant in the Universe after the photon; therefore, this indicates that the main source must be the cosmic beginning of the universe. Moreover, we know that neutrinos are well-known six in number with anti-neutrinos.

Furthermore, left- and right-handed versions of the fermions can be modelled in the standard model. Unfortunately, right-handed neutrinos have not been observed in the experiments. To solve this problem, the most conventional way is to add the right-handed neutrinos with very large Majorana masses to the standard Lagrangian. This is known as the See-saw mechanism, which denotes beyond of standard model. Theoretically, the See-saw mechanism requires the existence of heavy $\nu _{ R}$’s or other appropriate beyond Standard Model physics at very high energies, called the sterile neutrino. There are also some constraints on the mass scale of right-handed neutrinos as well as left-handed neutrinos^20,21^. It is assumed that the Majorana neutrinos with heavy mass should be close to the grand unified theory scale. In fact, experiments seem to hint at the possible existence of a fourth type of neutrino called a sterile neutrino that would interact even more rarely than the others. If neutrinos were their own antiparticles, they could have played a role in the early universe in expanding period. If neutrinos are their own antiparticles, neutrinoless double beta decay is possible. Such a process would favour matter over antimatter, creating an imbalance^22,23^. Additionally, neutrinoless double beta decay may imply that the neutrino masses could have a common origin^22,23^.

It should be stated that the physical properties of the neutrino have been experimentally investigated by using many defectors such as IMB, MACRO, Kamiokande II, Super-Kamiokande, SNO, LSND, MINOS, DONUT, CERN, OPERA, MiniBooNE, Fermilab, DUNE and KATRIN etc. Despite the significant achievements of neutrino physics, one can see that many open problems in this area arise from theory and experiments. For instance, although neutrinos have tiny masses, the absolute mass scale is still unknown. Neutrino oscillations are known to be sensitive only to the difference in the squares of the masses^24,25^. Different experiments measured neutrino masses, but the question of how neutrino masses arise has not been answered conclusively.

Theoretically, it is shown that the neutrino oscillations are periodic with a travelling distance *L* in space-time. Indeed, Pontecorvo’s theory of neutrino oscillation is based on Minkowski space-time. Neutrinos transform into each other due to transitions between mass and flavour eigenstates. However, this transition is not instantaneous but takes place at certain distances. Oscillation probabilities are defined for a certain critical distance. The probability and amplitude of oscillation are formalized in the Minkowski space-time framework, and the transition between eigenstates exhibits a Markovian character. Our study is currently looking at whether the oscillation period changes if neutrinos travel through deformed space-time. We know from fractional dynamics that such probabilities at the deformed space-times vary with the degree of deformation. In physics, there are numerous example of this. Gravitational perturbations can deform space-time. In this case, it can be expected that the oscillation dynamics deviate from Markovian to the non-Markovian since the deformed space-time leads to the memory effects in the jumping processes between the mass and flavour eigenstates. In this case, one can expect anomalous cyclic to appear in the neutrino oscillation. This behaviour can be described as a phase shift in the oscillation. It is important to note that the phase shift proposed by Ahluwalia and Burgard^26,27^. In this study, we will show that the phase shifting gradually increases in the subsequent oscillations. On the other hand, we will discuss the relationship between anomalous cyclic and neutrino relics.

The remaining organization of the study is as follows: before the fractional analysis of the neutrino oscillation, firstly, in “Theory of the neutrino oscillation”, we briefly summarize the solution of the time evolution of the Dirac equation and give the neutrino transition probability between different flavours. Then, in “Fractional dynamics between mass and flavour eigenstates”, we will obtain the solution of the fractional equations, and we will introduce a new transition probability which includes deformation of the space-time. We discussed the possible relation between anomalous cyclic of the neutrino oscillation and relic neutrinos in “Possible relation between anomalous cyclic and relic neutrinos”. Afterwards, we conclude the study with a discussion and conclusion in the last section.

## Theory of the neutrino oscillation

We summarize the mathematical background of the neutrino oscillation in the Minkowski space-time following the method in Ref.^28^. Dirac field theory is defined by introducing the following action

1 $$\begin{aligned} S = \int \bar{\psi }(t,\textbf{x}) (i\gamma ^{\mu } \partial _{\mu } -m ) \psi (t,\textbf{x}) \end{aligned}$$

which yields the following equation of motion

2 $$\begin{aligned} (i\gamma ^{\mu } \partial _{\mu } -m ) \psi (t,\textbf{x}) = 0 \end{aligned}$$

where $\gamma ^{\mu }$ are the Dirac-gamma matrices $(\mu =0,1,2,3)$, *m* is the mass of the particle and $\psi$ is the wave function of the spin-1/2 particle.

The flavour eigenstates of the neutrino denoted by $|\nu _{\alpha } \rangle$ can be represented as linear superposition of the mass eigenstates $|\nu _{j} \rangle$

3 $$\begin{aligned} |\nu _{\alpha } \rangle = \sum _{j} U^{*}_{\alpha j} |\nu _{ j} \rangle , \quad \alpha =e,\mu ,\tau , \quad j=1,2,3 \end{aligned}$$

where *U* is a unitary and non-diagonal mixing matrix that specifies the composition of each neutrino flavour state. *U* is given like Cabibbo–Kobyashi–Maskawa^29,30^ as

4 $$\begin{aligned} U=\begin{bmatrix} c_{1} &{} s_{1} c_{3} &{} s_{1} s_{3} \\ -s_{1} c_{2} &{} c_{1} c_{2}c_{3}-s_{2} s_{3}e^{i\delta } &{} c_{1} c_{2}s_{3}-s_{2} c_{3} e^{i\delta } \\ -s_{1} s_{2} &{} c_{1} s_{2}s_{3}-c_{2} s_{3}e^{i\delta } &{} c_{1} s_{2}s_{3}-c_{2} c_{3} e^{i\delta } \end{bmatrix} \end{aligned}$$

where $c_{i}=\cos \theta _{i}$, $s_{i}=\sin \theta _{i}$ and $\delta$ is the Dirac CP violating phase. This matrix is called as the Pontecorvo–Maki–Nakagawa–Sakata matrix^3,31^.

Conversely, by inverting the relation, we obtain the massive neutrino state as $|\nu _{j}\rangle = \sum _{j} U^{\dag }_{\alpha j} |\nu _{\alpha } \rangle$. The massive neutrino states are orthonormal as $\langle \nu _{j}|\nu _{k}\rangle =\delta _{jk}$. The mixing matrix has to be unitary transformation as $U^{\dag }U=UU^{\dag }=1$ which guarantees that $\sum _{j}U_{\alpha j}^{*}U_{\beta j}=\delta _{\alpha \beta }$ and $\sum _{\alpha }U_{\alpha j}^{*}U_{\alpha k}=\delta _{jk}$ implies that the flavour states are orthonormal as well, as was expected.

According to the standard approach, the propagation of mass eigenstate $|\nu _{j}\rangle$ evolve with time and it can be obtained by the solution of the Dirac equation in Eq. (2). The time evolution of the Dirac equation is given by

5 $$\begin{aligned} i \hbar \frac{\partial }{\partial t} \psi _{j} (t, \textbf{x}) = - \sqrt{\textbf{p}^{2} c^{2} + m^{2} c^{4} } \frac{ \mathbf {\sigma }\cdot \textbf{p} }{| \textbf{p} |} \psi _{j} (t, \textbf{x}) \end{aligned}$$

where $\psi _{j} (t, \textbf{x}) = \langle \textbf{x}|v_{j} \rangle$ and $\mathbf {\sigma }$ are Pauli matrices. In the ultra-relativistic limit, the Dirac equation (5) can be given as

6 $$\begin{aligned} i \hbar \frac{\partial }{\partial t} \psi _{j} (t, \textbf{x}) = - \left[ |\textbf{p}| c + \frac{m^{2}_{j} c^{3}}{2|\textbf{p}|} \right] \frac{ \mathbf {\sigma }\cdot \textbf{p} }{| \textbf{p} |} \psi _{j} (t, \textbf{x}) \end{aligned}$$

The solution of Eq. (6) can be obtained as

7 $$\begin{aligned} \psi _{j} (t, \textbf{x}) = e^{-i \frac{m^{2}_{j} c^{3}}{2 \hbar |\textbf{p}|} t} e^{-i\frac{pc}{\hbar } t} \end{aligned}$$

which is equivalent to

8 $$\begin{aligned} |\nu _{j} (t) \rangle = e^{-i \frac{m^{2}_{j} c^{3}}{2 \hbar |\textbf{p}|} t} e^{-i\frac{pc}{\hbar } t} |\nu _{j} \Big \rangle \end{aligned}$$

As a consequence of Eq. (3), we obtain

9 $$\begin{aligned} |\nu _{\alpha } (t) \rangle = \sum _{j} U_{\alpha j} e^{-i \frac{m^{2}_{j} c^{3}}{2 \hbar p} t} e^{-i\frac{pc}{\hbar } t} |\nu _{j} \Big \rangle \end{aligned}$$

The probability amplitude of the initial flavour eigenstate $|\nu _{\alpha } \rangle$ as another flavour eigenstate $|\nu _{\beta } \rangle$ is given by

10 $$\begin{aligned} \langle \nu _{\beta } |\nu _{\alpha } (t) \rangle = \sum _{j} U_{\alpha j} U^{*}_{\beta j} e^{-i \frac{m^{2}_{j} c^{3}}{2 \hbar p} t} e^{-i\frac{pc}{\hbar } t} \end{aligned}$$

Hence, the probability for a transition $|\nu _{\alpha } \rangle \rightarrow |\nu _{\beta } \rangle$ under time evolution is given by

11 $$\begin{aligned} P_{\nu _{\alpha } \rightarrow \nu _{\beta } } (t) = \sum _{jk} U_{\alpha j} U^{*}_{\beta j} U^{*}_{\alpha k} U_{\beta k} e^{-i \frac{( m^{2}_{j} - m^{2}_{k} ) c^{3}}{2 \hbar p} t} \end{aligned}$$

where we will set $t=L$.

**Figure 1:**
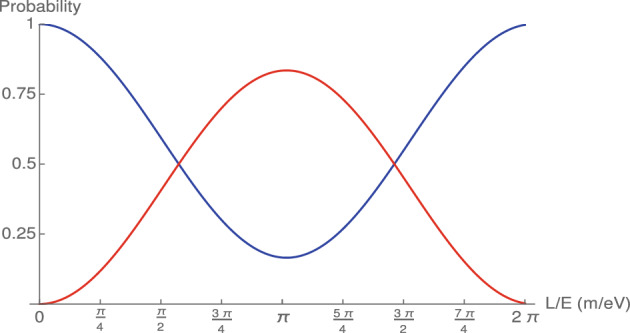
Neutrino oscillation probabilities assuming the initial state is an electron neutrino. The blue curve denotes the survival probability $P(\nu _{e} \rightarrow \nu _{e})$, the red curve corresponds to the transition probability from electron neutrino to a muon neutrino $P(\nu _{e} \rightarrow \nu _{\mu })$.

For example; to the probability of finding the neutrino with the muon flavour, Eq. (11) can be written in the form:

12 $$\begin{aligned} P(\nu _{e} \rightarrow \nu _{\mu }; t) = \sin ^{2}2\theta \sin ^{2} \left[ \frac{\Delta m^{2}}{4E} L \right] \end{aligned}$$

where *L* is the distance, *E* denotes the energy and $\Delta m^2$ involves the mass eigenstates since the flavour conversion happens when there is mixing between the mass eigenstates.

The oscillation probability obtained by Eq. (12) is plotted in the range of 0–$2\pi$ for two-flavour cases and given in Fig. 1. To show the periodicity of the oscillation, the horizontal axis is chosen as radial which corresponds to *L*/*E*. The horizontal axis represents the unit circle. In the numerical procedure we set $\theta _{12}=33^{0}$, $\Delta m^{2}_{12}=7.37\times 10^{-5}$ eV$^{2}$ where 1 denotes electron (or $\alpha$) and 2 indicates muon (or $\beta$). The parameter values used here are the best fit values for electron and muon neutrinos^32^.

In Fig. 1, we consider the initial state as electron neutrino. Blue curve present the survival probability of the electron neutrino which corresponds to $P(\nu _{e} \rightarrow \nu _{e})$. On the other hand, blue curve in the same figure represent transition probability from electron neutrino to the muon neutrino which is given as $P(\nu _{e} \rightarrow \nu _{\mu })$.

As can be seen from the figure, the oscillation is completed periodic in Minkowski space-time as expected in the theory. On the other hand, the survival probability of the electron neutrino $P(\nu _{e} \rightarrow \nu _{e})$ has maximum value at the points where the oscillation probabilities $P(\nu _{e} \rightarrow \nu _{\mu })$ are minimum. It is seen that almost nowhere the probability of oscillation and survival does not take values of zero or one. According to neutrino oscillation experiments, there is always the possibility of oscillation, and therefore, there is no case where the probability of oscillation is equal to zero and the probability of survival is equal to one.

In this schema, the oscillation length which is the characteristic length is inversely proportional to the squared mass $|\Delta m^{2}|$ and is linear with the neutrino energy *E*

13 $$\begin{aligned} L^{osc} = \frac{4\pi E}{ |\Delta m^{2}| } = 2.47 \frac{E}{ |\Delta m^{2}| } \frac{(\text {MeV})}{(\text {eV}^{2})} \,\text {m} = 2.47 \frac{E}{ |\Delta m^{2}| } \frac{(\text {GeV})}{(\text {eV}^{2})} \,\text {km} \end{aligned}$$

which gives a distance for a complete oscillation that corresponds to an important constraint on the measurement conditions. This constraint implies that the neutrino oscillation can be measured at the $L\sim L^{osc}$. At $L^{osc}$ the phase in Eq. (12) is generated by $\Delta m^{2}$ becomes equal to $2\pi$. To determine the oscillation length it is being considering an average energy of the neutrino flavours in some kind of experiments since neutrinos, for example from the Sun, emitted with different energies.

## Fractional dynamics between mass and flavour eigenstates

The neutrino oscillations between mass and flavour eigenstates in the Pontecorvo’s oscillation scheme is the periodic and Markovian for non-relativistic and relativistic neutrinos. The transition probability between neutrino flavours in Eq. (11) satisfies both the periodic and Markovian condition for the oscillation dynamics. It is well know in stochastic theory that the nature of the translation or oscillation dynamics are very strikingly depend on the structure of the space-time. The smallest change in the space-time structure significantly affects the dynamics of motion since dynamics is coupled to the space-time metric. In our case, there are many sources, such as gravitational waves and the mass distribution in the universe or decoherence and deformation of the Hilbert space at the quantum level^33^, that can cause the space-time deformations at both the local and general cosmological scales. Here, we consider the neutrino oscillation dynamics coupled to the space-time in the stochastic framework, however, the models where the oscillation dynamics explicitly coupled to gravity may also be interesting.

As a results, if we turn to the mathematical notation we say that in the classical and quantum levels the deformation in the space-time leads to divergent characteristic waiting time $T=\int _{0}^{\infty }dt w(t)t$ while the jump length variance $\Sigma ^{2}=\int _{-\infty }^{\infty } dx \lambda (x)x^{2}$ is finite where $w(t)=\int _{-\infty }^{\infty } dx \psi (x,t)$ and $\lambda (x)=\int _{0}^{\infty } dt \psi (x,t)$ are waiting time and the jump length probability distribution for $\psi (x,t)$ of particle wave function^34,35^. The non-Markovian motion has memory and leads to fractional dynamics^34–37^. Based on non-Markovian framework, Dirac equation in (6) for the deformed Minkowski space-time can be written^38–40^ as

14 $$\begin{aligned} i \hbar D_{t} ^{\eta } \psi _{j} (t, \textbf{x}) = - \left[ |\textbf{p}| c + \frac{m^{2}_{j} c^{3}}{2|\textbf{p}|} \right] \frac{ \mathbf {\sigma }\cdot \textbf{p} }{| \textbf{p} |} \psi _{j} (t, \textbf{x}) \end{aligned}$$

where $D_{t} ^{\eta }$ denotes the Caputo fractional derivative operator of order $\eta$ and $0<\eta <1$. Here with the aim of retaining the dimensional coherence on both sides of Eq. (14), the energy eigenvalue *E* varies with power $\eta$. For $\eta =1$, the fractional Schrödinger equation reduces to the standard one. The solution of Eq. (14) is the same form with Eq. (7). Therefore, Eq. (7) can be written as

15 $$\begin{aligned} \psi _{j} (t, \textbf{x}) = \chi ^{(0)}_{j} (t, \textbf{x}) \psi ^{(0)}_{j}(t,\textbf{x}) \end{aligned}$$

where

16 $$\begin{aligned} \chi ^{(0)}_{j} (t, \textbf{x}) = e^{-i \frac{m^{2}_{j} c^{3}}{2 \hbar |\textbf{p}|} t}, \qquad \psi ^{(0)}_{j}(t,\textbf{x}) = e^{-i\frac{pc}{\hbar } t}. \end{aligned}$$

Given wave functions in Eq. (16) satisfies

17 $$\begin{aligned} i D_{t} ^{\eta } \chi ^{(0)}_{j}(t,\textbf{x}) = - \lambda ^{\eta }_{1} \chi ^{(0)}_{j}(t,\textbf{x}), \quad \lambda _{1}= \frac{ c \mathbf {\sigma }\cdot \textbf{p} }{\hbar } \end{aligned}$$

and

18 $$\begin{aligned} i D_{t} ^{\eta } \psi ^{(0)}_{j}(t,\textbf{x}) = - \lambda ^{\eta }_{2} \psi ^{(0)}_{j}(t,\textbf{x}), \quad \lambda _{2} = \frac{m^{2}_{j} c^{3}}{2 \hbar |\textbf{p}|} \end{aligned}$$

In the Riemann–Liouville formalism, the fractional integral of order $\eta$ is given by the definition^34–37^

19 $$\begin{aligned} D_{t}^{\eta } * f(t) = \frac{1}{\Gamma (\eta )} \int _{0}^{t} \left( t-\tau \right) ^{\eta -1} f (\tau ) d\tau , \quad t>0 \end{aligned}$$

where $\eta$ is any positive number and $\Gamma (\eta )$ is Gamma function.

The solution of Eqs. (17) and (18) can be obtained by taking the Laplace transform. If we perform Laplace transformation on Eq. (17)

20 $$\begin{aligned} i \mathscr{L} \{ D_{t} ^{\eta } \psi ^{(0)}_{j}(t,\textbf{x}) \}= \mathscr{L} \{ \lambda ^ {\eta }_{1,2} \psi ^{(0)}_{j}(t,\textbf{x}) \} \end{aligned}$$

which yields

21 $$\begin{aligned} i s^{\eta } \tilde{\psi }^{(0)}_{j}(t,\textbf{x}) - i s^{\eta -1} \tilde{\psi }^{(0)}_{j}(0) = \lambda ^{\eta }_{1,2} \tilde{\psi }^{(0)}_{j}(t,\textbf{x}) \end{aligned}$$

where $\tilde{\psi }^{(0)}_{j}(t,\textbf{x})$ is the Laplace transform of the $| \nu _{k} (t) \rangle$, and it can be obtained as

22 $$\begin{aligned} \tilde{\psi }^{(0)}_{j}(t,\textbf{x}) = \tilde{\psi }^{(0)}_{j}(0) \frac{i s^{\eta -1} }{is^{\eta } - \lambda ^{\eta }_{1,2} } \end{aligned}$$

By employing the inverse Laplace transform one can obtain two independent solutions as

23 $$\begin{aligned} \chi ^{(0)}_{j}(t,\textbf{x}) = \chi ^{(0)}_{j}(0) E_{\eta } (-i \lambda ^{\eta }_{1} t^{\eta } ) \end{aligned}$$

and

24 $$\begin{aligned} \psi ^{(0)}_{j}(t,\textbf{x}) = \psi ^{(0)}_{j}(0) E_{\eta } (-i \lambda ^{\eta }_{2} t^{\eta } ) \end{aligned}$$

where $E_{\eta }$ denotes the Mittag–Leffler function^41^. It should be noted that the Mittag–Leffler function can be considered a generalization of the natural exponential one.

Combing Eqs. (23) and (24) into Eq. (15), general solution of fractional Dirac equation can be written as

25 $$\begin{aligned} \psi _{j}(t,\textbf{x}) = \psi ^{(0)}_{j}(0) E_{\eta } (-i \lambda ^{\eta }_{1} t^{\eta } ) E_{\eta } (-i \lambda ^{\eta }_{2} t^{\eta } ) \end{aligned}$$

As a consequence of Eq. (3), we obtain

26 $$\begin{aligned} | \nu _{j} (t) \rangle = \sum _{j=1}^{3} U_{\alpha j} E_{\eta } (-i \lambda ^ {\eta }_{1} t^{\eta } ) E_{\eta } (-i \lambda ^ {\eta }_{2} t^{\eta } ) | \nu _{j} \rangle \end{aligned}$$

The series expression of the Mittag–Leffler function is given as

27 $$\begin{aligned} E_{\eta } (-i \lambda ^ {\eta } t^{\eta } ) = \sum _{j=0}^{\infty } \frac{(-i \lambda ^ {\eta } t^{\eta })^{j} }{\Gamma (1+\eta j)} \end{aligned}$$

For $\eta =1$, Eq. (27) reduces to the standard exponential form, whereas for $0<\eta < 1$, initial stretched exponential behaviour

28 $$\begin{aligned} E_{\eta } (-i \lambda ^ {\eta } t^{\eta } ) \sim \exp \left( - \frac{i \lambda ^ {\eta } t^{\eta } }{\Gamma (1+\eta )} \right) \end{aligned}$$

turns over to the power-law long-time behaviour

29 $$\begin{aligned} E_{\eta } (-i \lambda ^ {\eta } t^{\eta } ) \sim \frac{1 }{i \Gamma (1-\eta )\lambda ^ {\eta } t^{\eta }}. \end{aligned}$$

By using the above expression, we can give Eq. (26) in the simplified form

30 $$\begin{aligned} | \nu _{\alpha } (t) \rangle = \sum _{j=1}^{3} U_{\alpha j} e^{-i \left( \frac{m^{2}_{j} c^{3}}{2 \hbar p} \right) ^{\eta } t^{\eta } } e^{ -i \left( \frac{pc}{\hbar }\right) ^{\eta } t^{\eta } } | \nu _{j} \rangle \end{aligned}$$

In this case, the probability amplitude becomes

31 $$\begin{aligned} \langle \nu _{\beta } |\nu _{\alpha } (t) \rangle = \sum _{j} U_{\alpha j} U^{*}_{\beta j} e^{-i \left( \frac{m^{2}_{j} c^{3}}{2 \hbar p} \right) ^{\eta } t^{\eta } } e^{ -i \left( \frac{pc}{\hbar }\right) ^{\eta } t^{\eta } } \end{aligned}$$

Hence, the probability for a transition $| \nu _{\alpha } \rangle \rightarrow | \nu _{\beta } \rangle$ under time evolution is

32 $$\begin{aligned} P_{\nu _{\alpha } \rightarrow \nu _{\beta } } (t) = \sum _{jk} U_{\alpha j} U^{*}_{\beta j} U^{*}_{\alpha k} U_{\beta k} e^{-i \left( \frac{(m^{2}_{j} - m^{2}_{k} ) c^{3}}{2 \hbar p}\right) ^{\eta } t^{\eta } } \end{aligned}$$

where we will set $t=L$. One can see that the transition probability has the stretched exponential form which is often called as the Kohlrausch–Williams–Watts function^42,43^. It should be noted that for $\eta =1$, Eq. (32) reduces to Eq. (11).

**Figure 2:**
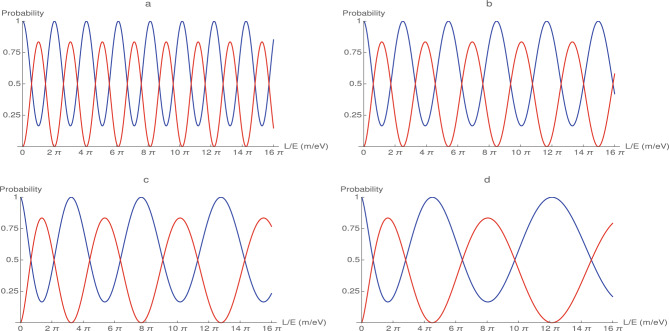
As in Fig. 1, neutrino oscillation probabilities assuming the initial state is an electron neutrino. The blue curve denotes the survival probability $P(\nu _{e} \rightarrow \nu _{e})$, the red curve corresponds to the transition probability from electron neutrino to a muon neutrino $P(\nu _{e} \rightarrow \nu _{\mu })$. In (**a**), the survival and oscillation probability in the Minkowski space-time for $\eta =1$ indicates the ordinary Minkowski space-time. The oscillation probabilities in the deformed Minkowski space-time are given in (**b**–**d**) sub-figures. The deformation parameters of the Minkowski space-time are $\eta =0.9$ in (**b**) $\eta =0.8$ in (**c**) and $\eta =0.7$ in (**d**).

Here, we analytically obtained neutrino oscillation probability for the deformed Minkowski space-time above. To see the effect of the fractional order on the survival and oscillation probability, assuming the initial flavour as electron neutrino, we numerically solved Eq. (32) for various $\eta$ values and plotted all numerical results in Fig. 2. As in Fig. 2 as, in the numerical procedure we set $\theta _{12}=33^{0}$, $\Delta m^{2}_{12}=7.37\times 10^{-5}$ eV$^{2}$ and $c=3\times 10^{8}$ m/s. As mentioned above, these parameters are obtained by fitting experimental results between electron and muon oscillations^32^.

In Fig. 2a–d, the blue curve denotes the survival probability of the electron neutrino $P(\nu _{e} \rightarrow \nu _{e})$ and the red line indicates the oscillation probability transition from electron neutrino to the muon neutrino $P(\nu _{e} \rightarrow \nu _{\mu })$.

In Fig. 2a, oscillation probability in the Minkowski space-time for $\eta =1$ which indicates ordinary Minkowski space-time. Therefore, the solution in this figure are the same with Fig. 2. However, the survival probability of electron neutrino and oscillation probabilities electron neutrino to the muon neutrino for the fractional Minkowski space-time are given in Fig. 2b–d sub-figures. The fractional parameters of Minkowski space-time are set as $\eta =0.9$ in (b) $\eta =0.8$ in (c) and $\eta =0.7$ in (d).

As can be seen from Fig. 2, the survival probability of the electron neutrino $P(\nu _{e} \rightarrow \nu _{e})$ has maximum value at the points where the oscillation probabilities $P(\nu _{e} \rightarrow \nu _{\mu })$ are minimum. It is seen that almost nowhere the probability of oscillation and survival does not take values of zero or one as well in Fig. 1.

As mentioned above the survival probability and oscillation probability remain the same without change for $\eta =1.0$ which corresponds to the Minkowski space-time where the oscillations are quite smooth and appear subsequently at the transition points $T_{p} = \pi , 3\pi , 5\pi ,\ldots$ as seen from Fig. 2a or Table 1. However, the neutrino survival and oscillation probability for the deformed Minkowski space-time is quite different as can be seen from Fig. 2b–d unlike for $\eta =1$ as well as in Fig. 1 or Fig. 2b. However, for $\eta < 1$ the survival and oscillation probabilities in the deformed Minkowski space leads to two main and important results:

Firstly, as it can be seen from Fig. 2a or Table 1 the survival probability of electron neutrino $P(\nu _{e} \rightarrow \nu _{e})$ and oscillation probability transition from electron neutrino to the muon neutrino $P(\nu _{e} \rightarrow \nu _{\mu })$ takes the maximum and minimum values $T_{p} = \pi , 3\pi , 5\pi ,\ldots$ for $\eta =1$, respectively. However, for $\eta < 1$, the survival and transition points slide to the right and these increments regularly increase for the subsequent oscillations as seen in Fig. 2b–d. This means that when $\eta$ decreases, the period of oscillation shifts. For $\eta < 1$, the amounts of the increments in the survival and transition points are given in Table 1. One can see that the shifts in the survival and transition points increase when $\eta$ values decrease. These results strongly provide that the fractional dynamics between mass and flavour eigenstates lead to anomalous behaviour in neutrino survival and oscillation probabilities.

Secondly, the period length of the oscillation between two neutrino flavour, for instance electron and muon neutrinos, i.e., oscillation time, regularly increases for each $\eta$ value depending on the shifts of the transition points. This behaviour may be interpreted as gravitational red and blue shifts^44^. This behaviour can also be seen in Fig. 2b–d. One can see that the period lengths dramatically increase when $\eta$ decreases. The increments in the period length are given Table 2. These results clearly indicate that deformation in space-time extends the survival probabilities of the flavours. On the other hand, we expect that if a particle whose period length changes in a deformed Minkowski space-time continues to move in a homogeneous Minkowski space-time, it is expected that the particle will return to its original period-length state and can continue to oscillate in its original form. Furthermore, our results provide that the oscillation length in the deformed Minkowski space-time is bigger than $L^{osc}$ i.e., $L>>L^{osc}$.

**Table 1:** Oscillation points for various $\eta$ values.

$\eta$	$T_{p1}$	$T_{p2}$	$T_{p3}$	$T_{p4}$
$\eta =1.0$	$\pi$	$3\pi$	$5\pi$	$7\pi$
$\eta =0.9$	$1.11\pi$	$3.88\pi$	$6.90\pi$	$0.02\pi$
$\eta =0.8$	$1.38\pi$	$5.34\pi$	$10.17\pi$	$15.47\pi$
$\eta =0.7$	$1.67\pi$	$8.05\pi$	$16.62\pi$	$27.04\pi$

**Table 2:** The period lengths of the oscillation for various $\eta$ values.

$\eta$	$l_{1}$	$l_{2}$	$l_{3}$	$l_{4}$
$\eta =1.0$	$2\pi$	$2\pi$	$2\pi$	$2\pi$
$\eta =0.9$	$2.50\pi$	$2.88\pi$	$3.04\pi$	$3.19\pi$
$\eta =0.8$	$3.22\pi$	$4.49\pi$	$5.14\pi$	$5.47\pi$
$\eta =0.7$	$4.52\pi$	$7.59\pi$	$9.50\pi$	$10.95\pi$

## Possible relation between the anomalous cyclic and relic neutrinos

Now, we discuss the possible link between anomalous behaviour of the neutrino oscillation and the relic neutrinos. It is known that at the cosmic beginning of the Universe, the neutrinos separated from the matter at the $t=1$ s. At this stage, the majority of neutrinos should have been produced after the photon. This is called the cosmic neutrino background (CNB or C$\nu$B). In the theoretical calculations, it is estimated that C$\nu$B has a temperature of approximately $T \approx 1.95$ K. The relic neutrinos are the most important phenomenon like the Cosmic Microwave Background (CMB). As is known, the CMB is another big remnant that appears at around 380,000 years after the cosmic beginning, and its temperature was determined to be approximately $T \approx 2.725$ K. It is known that the C$\nu$B is screened by the CMB due to its low energy.

Observing the relic neutrinos is of great value in confirming theoretical predictions of particle physics. It is also of great importance to understand the role of relic neutrinos in the universe. However, relic neutrinos have not been able detected directly or indirectly until now. There are two main reasons for this: (1) the C$\nu$B temperature is lower than the CMB temperature, (2) they weakly interact with matter. Therefore, the direct^45^ and indirect methods^46^ are proposed to observe or detect the neutrino relic. However, no successful observation has yet been achieved so far. For example, one of the methods is to analyse the images taken from the Planck data. The energies of the cold regions in these data images are estimated to be $T \approx 1.95$ K and it is supposed that these regions may evidence for the relic neutrinos. However, these observational findings were not accepted as sufficient evidence for the relic neutrinos. Maybe, James Webb telescope and other satellites in future can provide new and more detailed information on this background image. On the other hand, the relic neutrinos are tried to be observed in direct particle experiments. Weinberg proposed a method to directly detect the relic neutrinos^45^. According to this scenario, if a neutrino can be captured on a tritium target^45^ on the process: $\nu + {}^3$H $\rightarrow {}^3$He $+ e^{-}$, this captured neutrino is very likely to be a relic neutrino which has the low-energy. To detect such kind of neutrinos, many methods and experimental setups have been proposed so far, for example, KATRIN^47^, PTOLEMY^48,49^ etc. It is known that KATRIN and PTOLEMY are operating to capture relic neutrinos over the particle process. In these experimental studies, new constraints to captured relic neutrinos have been defined in details^47–49^. For the electron based neutrinos the neutrino mass square is obtained as $\Delta m^{2} < 1.1$ eV at 90% C.L. The limit come from the kinematics of $^3$H $\beta$ decay derived from the first month of data collected by the KATRIN tritium endpoint experiment^47,50^. However, as a result, despite all these efforts, the relic neutrinos have not been able detected experimentally or observationally.

Here we consider a possible relation between the anomalous cyclic and relic neutrinos. It is known that the relic neutrinos are also the left-handed electron, muon, and tau neutrinos, and their anti-neutrinos belong to the standard neutrino family. Their main feature is that they have low-energy, that is, non-relativistic, and that they emerged at the beginning of the universe. According to the theoretical scheme, these neutrinos, like all other neutrinos, oscillate between the mass and flavour eigenstates. The main difference between relic neutrinos and neutrinos produced from other sources is that they are very old and have been travelling through space-time for about 13.8 billion years. As we suggest in this study, if a phase shift is possible in neutrino oscillation, the most phase shift should have occurred in relic neutrinos that are about 13.8 billion years old. From this perspective, it is a remarkable suggestion to establish a relationship between relic neutrinos and anomalous cycles. This idea implies that there is a relationship between the age of neutrinos and the phase shift in oscillation. It is possible to say that the neutrinos with the most phase shifts in the universe are relic neutrinos.

As can be seen from Figs. 1 and 2a, the oscillation periodicity does not depend on the value of the *L*/*E* ratio. Unlike Figs. 1 and 2a, Fig. 2c,d confirm that the phase shift strongly depends on the parameter $\eta$. For $\eta <1$ values, when L/E increases, the phase shift in the oscillation also increases steadily. Therefore, it is expected that the oscillation length of the relic neutrinos must be very large when compared to other younger neutrinos. Although relic neutrinos are low-energy neutrinos, various scenarios can be developed to observe them on the cosmological scale. However, the primary problem at the moment is to observe the phase shift in the oscillation.

## Discussion and conclusion

The standard theory of neutrino oscillation for the Minkowski space-time was briefly summarized in this study. In Fig. 1, we plotted the oscillation probability. Then, we performed a new oscillation probability function for the neutrinos for the deformed Minkowski space-time and presented the numerical results in in Fig. 2. We demonstrate that anomalous cyclic behavior in the neutrino oscillation can appear in the deformed Minkowski space-time. Furthermore, we explored the possible relationship between the anomalous cyclic behavior of neutrinos and relic neutrinos.

As we know that in the standard neutrino theory, the phase shift does not appear in the oscillation probability as seen in Eq. (11). However, the fractional parameter $\eta$ in Eq. (32) causes the oscillation period to stretch. As we mentioned in “Introduction” and “Fractional dynamics between mass and flavour eigenstates”, to appear the non-Markovian dynamics in neutrino oscillation, it does not need to coupling of the gravity with the mass and flavour eigenstates. It is sufficient for the space-time metric to be deformed for the emergence of non-Markovian dynamics. The most suitable candidates for this are local and non-local gravitational waves and perturbations. In the stochastic theory, this situation is due to the fractional nature of space-time. However, in this study, we only considered time-fractional dynamics for simplicity. On the other hand, one can see from literature that phase shift problem in the neutrino oscillation has been studied by using different mechanisms^26,27,51,52^.

At this point, furthermore, it should be noticed that this discussion is valid only for neutrinos. The theoretical approach we present here applies only to neutrinos that change flavour by switching between mass and flavour eigenstates. It cannot be applied to other elementary particles. As is known other elementary particles—except the neutrinos—do not oscillates^53,54^ in a similar way since they have not mass and flavour eigenstates like neutrinos. However, this discussion can be generalized to the Friedman–Robertson–Walker space-time, expanding universe or curve space-times^55^.

We have also noticed that the CP and T symmetries are violate in the neutrino oscillation as $P(\nu _{\alpha } \rightarrow \nu _{\beta }) \ne P( \bar{\nu }_{\alpha } \rightarrow \bar{\nu }_{\beta })$ and $P(\nu _{\alpha } \rightarrow \nu _{\beta }) \ne P( \nu _{\beta } \rightarrow \nu _{\alpha })$, respectively. However, it is not easy to see CP and T violation from the exponential form of the oscillation probability in Eqs. (11) or (32). To see these violations, it may be sufficient to look at their trigonometric forms of Eqs. (11) or (32) which contain *cos* and *sin* forms. For example, one can replace the matrix *U* with its complex conjugate $U^{*}$ in Eqs. (11) or (32), in this case, the *sin* terms change sign while the *cos* terms remain invariant, which denotes CP violation $P(\nu _{\alpha } \rightarrow \nu _{\beta }) \ne P( \bar{\nu }_{\alpha } \rightarrow \bar{\nu }_{\beta })$. On the other hand, the oscillation probability under interchanging of indices $\alpha \leftrightarrow \beta$ can change since under this operation the sign of *sin* terms change, which corresponds to violation of T reversal symmetry $P(\nu _{\alpha } \rightarrow \nu _{\beta }) \ne P( \nu _{\beta } \rightarrow \nu _{\alpha })$. However, it is easy to see that under both $U\rightarrow U^{*}$ and $\alpha \leftrightarrow \beta$ transformations in Eq. (32) remains invariant. Therefore, in this case of replacement of $\nu$ with $\bar{\nu }$ and the interchange of the initial and final flavours, the CPT is preserved as $P(\nu _{\alpha } \rightarrow \nu _{\beta }) = P( \bar{\nu }_{\beta } \rightarrow \bar{\nu }_{\alpha })$ in the deformed space-time. On the other hand, when $\alpha =\beta$ is chosen, CP and T violating effects do not exist for the diagonal transitions ($\nu _{e} \rightarrow \nu _{e}$, $\nu _{\mu } \rightarrow \nu _{\mu }$, $\nu _{\tau } \rightarrow \nu _{\tau }$). As a results, CPT symmetry cannot be violated and the oscillation dynamics is also Lorentz invariant as excepted. Additionally, it would be appropriate to draw attention to another crucial point that when $\Delta m^{2}_{ij} \rightarrow -\Delta m^{2}_{ij}$, the oscillation probabilities in Eqs. (11) or (32) remain the same since the mixing angle will change as $\theta _{ij} \rightarrow \pi /2-\theta _{ij}$ that leads to redefining of the mass eigenstates. This imply that the oscillation probability is also invariant under such transformation.

Obtained theoretical results in the present work clearly show that the phase shift in the neutrino oscillation can appear depend on deformed space-time and the phase shift in the oscillation period increases with time like photon. As we mentioned in abstract that the modification in the oscillation probabilities due to the studied effect in this work could be seen using relic neutrinos Another say, this theoretical results can be used to detect the relic neutrinos, vice versa. Although it is very difficult to detect the cyclic anomalies in the neutrino oscillations at the present particle detectors, it may not be impossible. Indirect observations can mediate us to observe the oscillation anomaly. For example, to detect phase shifts in neutrino oscillation, it can be analysed changing in the measured neutrino flux on Earth at the moment when the gravitational waves are observed after the black holes, neutron star collisions, or supernova explosions. Observation of a correlation between rippling gravitational waves and neutrino flux may be indirect evidence for phase shifting in neutrino oscillation.

## Data Availability

The datasets used and analysed during the current study available from the corresponding author on reasonable request.
